# Monitoring HIV Treatment and the Health Sector Cascade: From Treatment Numbers to Impact

**DOI:** 10.1007/s10461-017-1754-1

**Published:** 2017-04-11

**Authors:** Daniel Low-Beer, Michel Beusenberg, Chika Hayashi, Txema Calleja, Kimberly Marsh, Awandha Mamahit, Theresa Babovic, Gottfried Hirnschall

**Affiliations:** 1 HIV Department, WHO, Geneva, Switzerland; 2 UNAIDS, Geneva, Switzerland

**Keywords:** HIV, Treatment, Health sector cascade, Sustainable development goals, Impact, Program improvement

## Abstract

Although not originally part of the MDGs, HIV treatment has been at the center of global HIV reporting since 2003, marked by achievement of the target of 15 million people receiving treatment before 2015 and 18.2 million (16.1–19.0 million) by mid 2016. Monitoring of treatment has been strengthened with harmonized partner reporting and accountability with regular, annual reports. Beyond treatment numbers, increasingly measures of treatment adherence, retention and outcomes have been reported though with varying quality and completeness. However, with the sustainable development goals (SDGs), monitoring treatment is changing in three important ways. First, treatment monitoring is shifting from numbers to coverage and gaps in a cascade of services to achieve universal access. Secondly, this requires greater emphasis on disaggregated, individual level patient and case monitoring systems, which can better support linkage, retention and chronic, long term care. Thirdly, the prevention, testing and treatment cascade with a clear results chain, links treatment numbers to impact, in terms of reduced viral load, mortality and incidence. This agenda will require a greater contribution of routine impact evaluation alongside monitoring, with treatment seen as part of a cascade of services to ensure impact on mortality and incidence. In conclusion, the shift from monitoring treatment numbers to treatment linked to universal access to prevention, testing and treatment and impact on mortality and incidence, will be critical to monitor, evaluate, and improve HIV programs as part of the SDGs.

## Introduction

In 2000, HIV treatment monitoring and targets were not at the center of the UN General Assembly Resolution on AIDS [1]. There was divided commitment to global treatment, and despite over 500,000 people on treatment globally, only 11,000 received ARVs in Africa [2]. With the support of civil society and government leaders, together with new funding sources, PEPFAR and the Global Fund to fight AIDS, TB and malaria, treatment numbers and targets became central to HIV global monitoring.

Treatment monitoring was supported by three key components, explicit global targets, harmonized reporting among partners through UNGASS, the 3 by 5 initiative, and the Global AIDS Response Progress Reporting Process (GARPR), and finally regular accountability at global and country levels, with treatment targets set and used by donors and countries. There were challenges in reporting treatment outcomes, including retention and data on mortality reductions, and in overlap of reporting between donors.

This paper reviews the progress in treatment monitoring and results from 2000 to 2015, and the challenges of monitoring for the SDGs in the light of new global goals, treatment and strategic information guidelines.

## Developing Global Treatment Goals, 2000–2015

In the very first few years following UNGASS [1], there were no agreed global targets for treatment, and similarly access to treatment was also not global and did not for example address the treatment needs in Africa or low-income countries.

**Fig. 1:**
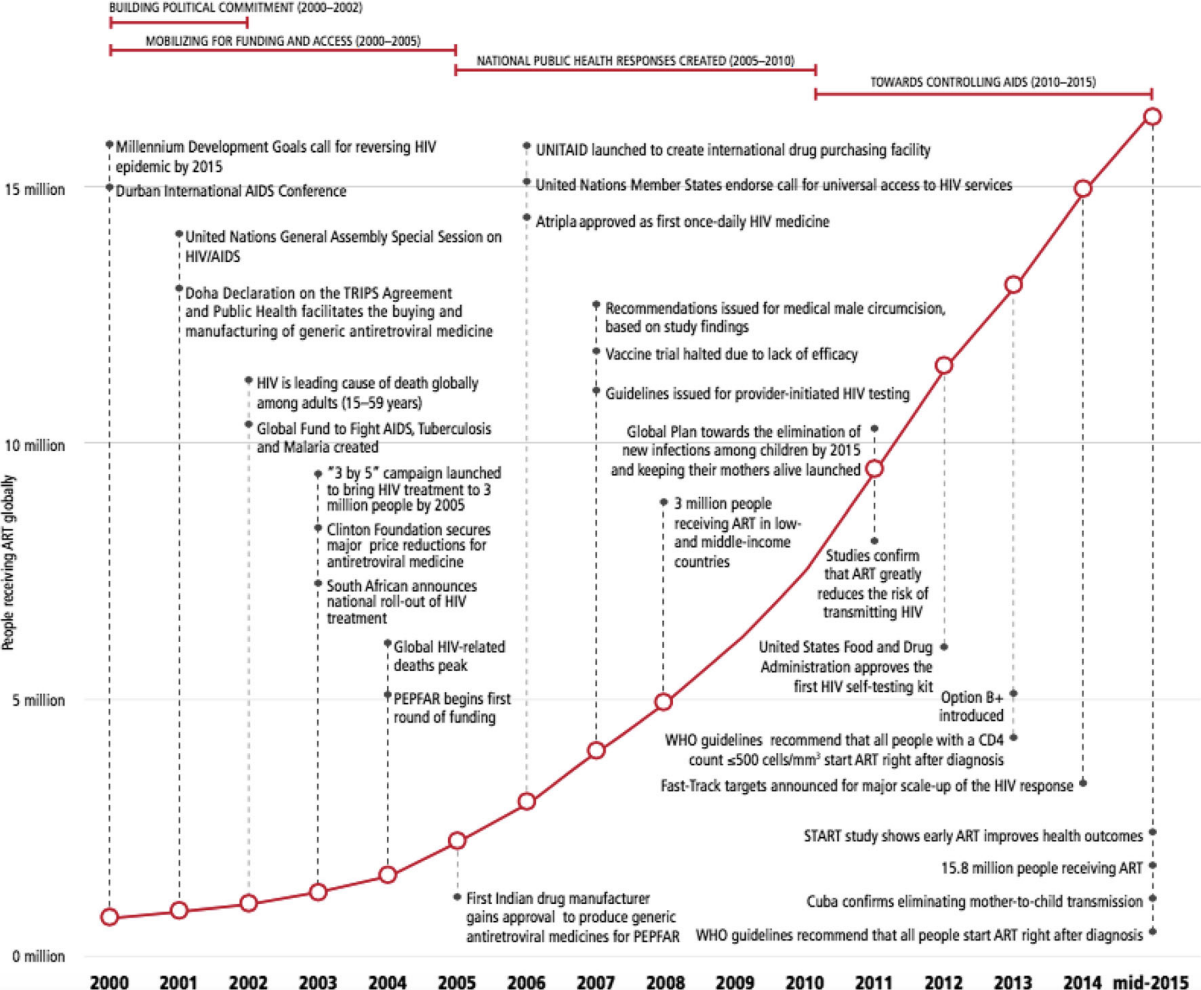
Timeline showing key events versus the number of people receiving antiretroviral therapy, 2000–2015

There have then been a number of stages in the development of treatment monitoring as shown in Fig. 1: an initial period of building political commitment (2000–2002) followed by the setting of global goals and building targets into national treatment programs (from 2003 to 2010), and most recently by a period of closing the treatment gap as part of controlling AIDS (2010–2015) [2, 3].

The “3 by 5” program launched by WHO in 2003 set the first global targets of 3 million people on treatment by 2005 [4]. This initiative was supported with technical assistance to countries in setting targets and strengthening strategic information systems to report on them.

In the same year, major price reductions in antiretroviral medicines were negotiated by partners and countries. South Africa announced the national roll-out of HIV treatment, which despite initial delays, has now become the largest global treatment program, with over 3 million people on treatment in that country [2]. However the global target of 3 million people receiving ART in low and middle-income countries was not reached until 2008.

Despite the challenges in reaching the 3 by 5 target, by 2006 United Nations Member States called for and endorsed calls for universal access to HIV services, including treatment [5]. This was supported by the launch of financing initiatives from PEPFAR and the Global Fund, and the emergence of Indian drug manufacturers, which gained approval to produce generic ARVs for PEPFAR from 2005.

At the international level, WHO was committed since 2006 to monitor and report on global progress in countries’ health sector responses towards universal access to HIV prevention, treatment, care and support, and brought together a broad set of 39 indicators [6].

After the 2011 Political Declaration on HIV/AIDS, UNAIDS, WHO and UNICEF developed GARPR—the joint online reporting tool, which was comprised of Global AIDS response progress indicators as well as WHO/UNICEF Universal Access indicators [7].

A common set of indicators related to antiretroviral treatment is listed below with the year in which data for these indicators were collected for the first time.

- People receiving antiretroviral therapy (2002)
- Twelve-month retention on antiretroviral therapy (2007)
- Twenty-four retention on antiretroviral therapy (2007)
- Sixty-month retention on antiretroviral therapy (2010)
- Health facilities that offer antiretroviral therapy (2007)
- ARV stock-outs (2007)
- HIV care (2007)
- Viral load (2014)

Retention indicators measure progress in increasing survival among infected adults and children by maintaining them on antiretroviral therapy. These data can be used to demonstrate the effectiveness of those programmes and highlight obstacles to expanding and improving them. The stock-out indicator measures a key aspect of antiretroviral drug supply management: whether health facilities dispensing ARV drugs have run out of stock of at least one required drug in the last 12 months. In addition, information on policy questions on the uptake of WHO recommendations were collected as well as treatment costs in a regular ARV survey.

The completeness of reporting by countries on treatment-related indicators was high, in 2015, 135 of 144 low- and middle-income countries (LMIC) provided data on access to ART, accounting for 96% of people receiving treatment. The response rate for number of ARV facilities was almost the same (134 LMIC in 2015), followed by ARV stock-outs and 12 months retention (both at 108 LMIC in 2015) and adults and children enrolled in care (94 LMIC in 2015). The other indicators had significantly lower response rates. There also remain challenges and limitations to reporting of retention and stock outs, and improved triangulation between drug, diagnostic and clinical data are required. The challenges include over and under reporting due to missing or delayed facility reports, the challenges of aggregating across treatment registers, and removing duplication of people from registers who have disengaged from care or transferred between facilities.

Over time more age disaggregations were added to core indicators such as people receiving ART. This included tracking of child infections and children on treatment.

In 2014, GARPR added a feature to enable reporting on people receiving ART at a subnational level, as data at national level do not capture differences in progress at lower geographic levels. The sub-national indicator offers an opportunity to improve program focus and planning at lower geographic levels.

**Fig. 2:**
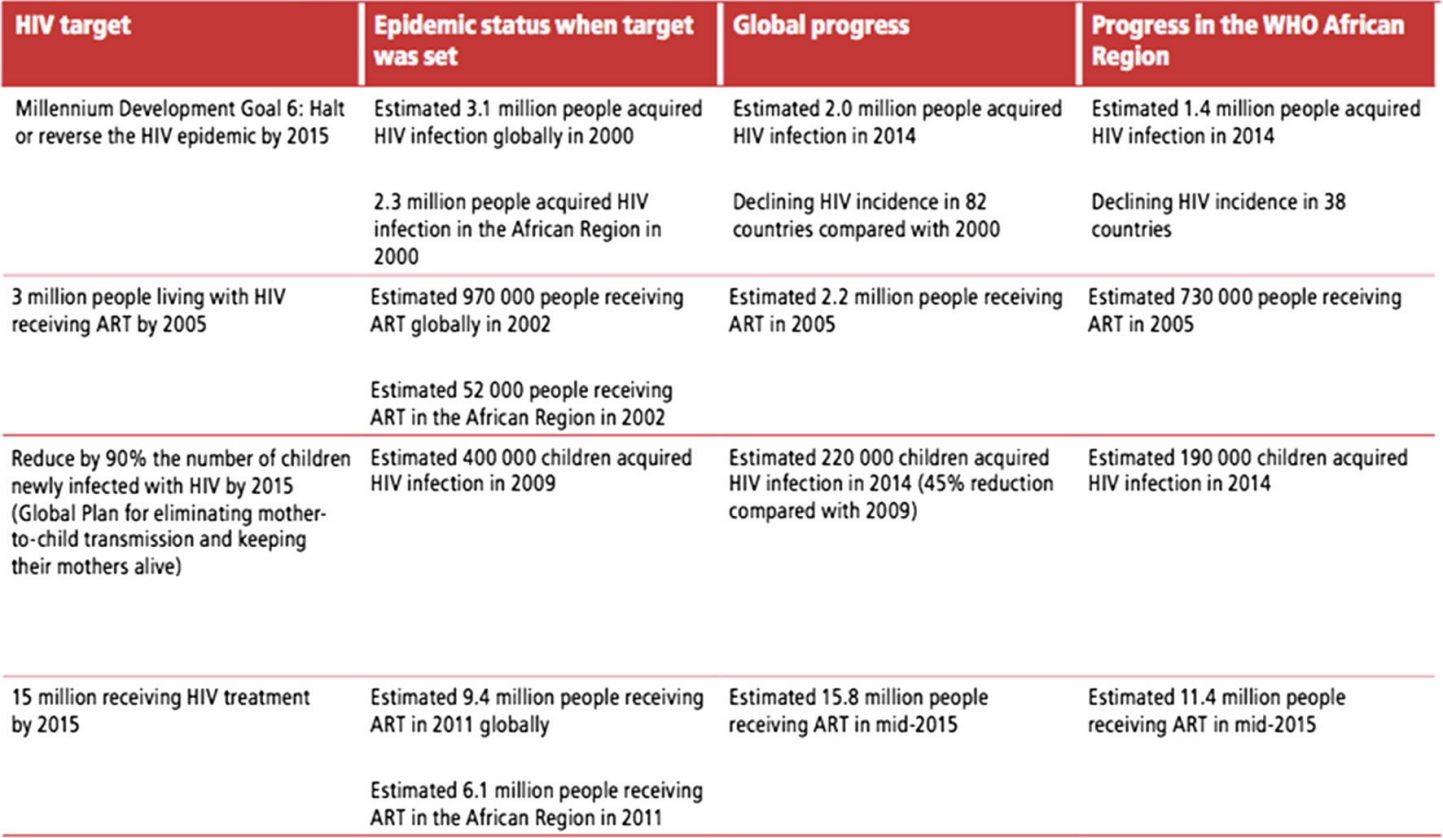
Progress made against key global targets, 2000–2015

During the period to 2015, key targets were set for treatment, as shown in Fig. 2, treatment data was harmonised between partners on an annual basis, and global, regional and country progress reported regularly.

A key component in the development of treatment monitoring and targets was the changing eligibility for HIV treatment, as defined by successive treatment guidelines from 2003 to 2015.

Initially treatment was focused on eligibility at late stage disease to reduce deaths. The recent 2015 guidelines, based on the results of studies showing the benefits of early treatment, now suggest all people who are living with HIV should be eligible for treatment [8, 9]. The denominator for targets, which was previously those below a certain CD4 count threshold and changed over time, is therefore now all people living with HIV [10].

## Global Treatment Monitoring and Results

A number of key components have been identified which drove the setting, accountability and progress towards treatment targets [2]:

1. Political commitment and partnerships were focused on targets—raising financing and promoting accountability for these targets.
2. A public health approach was put into practice—the targets were supported by a package of HIV services and guidance.
3. Civil society extended the HIV response into communities—civil society advocated for global targets, reduced treatment prices, and supported the expansion of services into communities.
4. Funding was mobilized and costs were reduced—global commitments were followed through with financing partnerships, domestic funding also increased substantially, and treatment costs reduced by 90%.
5. Innovations in science and implementation were widely used—global technical and implementation guidance was adapted over time as new science and implementation experience was harnessed.
6. Data was improved and increasingly drove decisions—targets were set at global and country levels and supported by investments in data, so that global targets went together with investment in country M&E systems.

In terms of data, there were important developments during this period, which supported treatment targets, accountability and monitoring.

First, UNGASS/GARPR reporting was developed where governments and partners shared their HIV data in a standardized manner, enabling comparable data to be reported [1]. By 2015, 180 countries were reporting their HIV program results in a consistent manner. These kinds of accountability mechanisms were relatively unique in the development sector, and supported global HIV and treatment reporting in an aligned manner.

Secondly, treatment targets were incorporated into national strategies, and into the investment models of partners, for example PEPFAR and the Global Fund. Progress against the targets was regularly and widely reported, together with the analysis of progress and areas for improvement, often including stakeholders for example from civil society.

Thirdly, there was an explicit commitment to investment in country data systems, generally with guidance that 5–10% of program funds be used to strengthen M&E systems [10]. This included significant commitments from PEPFAR to support population based surveys (increasingly featuring periodic biological markers), clinical and facility data systems, electronic district health information systems and key population mapping, strategic initiatives to invest in data from the Global Fund, and deployment of strategic information officers by UNAIDS, WHO and other partners. Increasingly treatment data was disaggregated geographically to sub national and facility levels, and linked to the district health information system such as the DHIS 2. However the focus of investments and accountability was on verifying treatment numbers, and gaps remain in adherence and impact data. Country data systems will require considerable investments to strengthen individual level patient monitoring to support chronic care to people with HIV over their lifetime. This will require greater attention to achieve the people centered reporting and monitoring systems for the SDGs.

As important was the investment in the demand for data, with regular country program and epi reviews, and performance used as a basis for funding by the Global Fund and increasingly by PEPFAR.

Nevertheless the focus on a single treatment target and number, underlined the rapid increase in access to treatment to 2015, shown in Figs. 1 and 2. The results were harmonized between partners, which meant treatment numbers were one of the most complete and consistent areas of HIV reporting. However there were gaps in terms of treatment quality and outcomes [11]. There are also some uncertainties in treatment data, and triangulation is required with drug and procurement data and further investments in patient monitoring systems based on the use of unique identifiers.

The target of 15 million people receiving treatment was achieved in advance of the deadline for 2015, with 18.2 million (16.1–19.0 million) on treatment as of mid-2016 [1]. Distinctively for a health and development issue, coverage of treatment is greater in Eastern and Southern Africa by 2015 than other regions, which is a remarkable achievement. People with HIV are more likely to receive ARVs in Eastern and Southern Africa than in other regions. However there remain major gaps in coverage by region, notably in West and Central Africa, and in particular among men who require health services to reach beyond the health facility. With the release of new WHO treatment guidelines in 2015 promoting Treat All [8], this shows some of the limitations of a single numerical target, and the need to identify gaps in coverage and the impact of treatment.

**Fig. 3:**
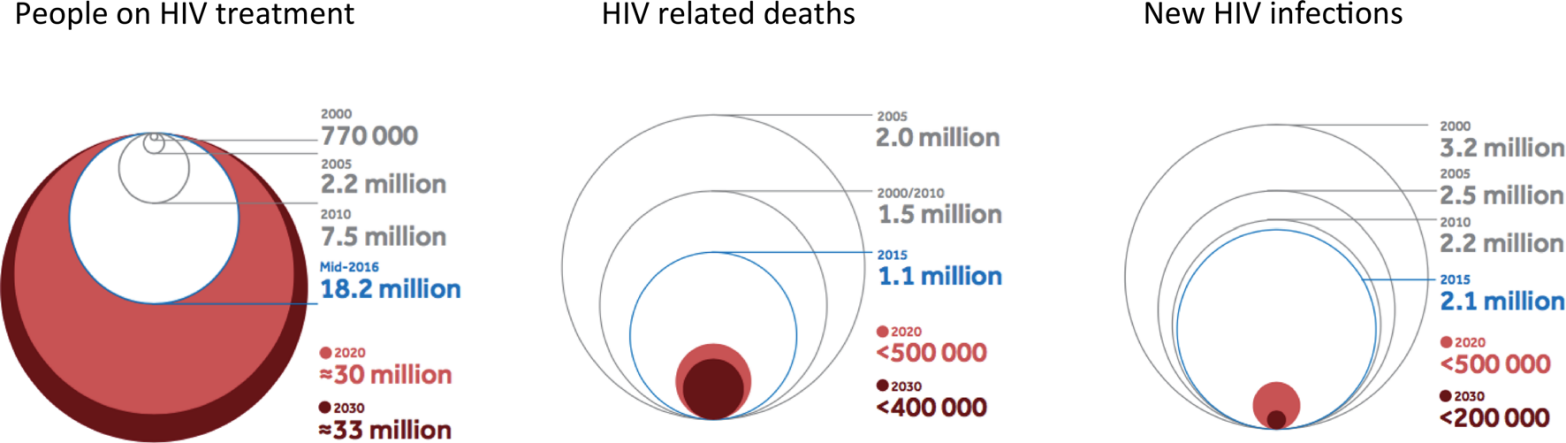
Progress in the global HIV response for people on ARVs, HIV related deaths and new HIV infections and targets to 2030 (*red shading* shows future targets, *blue line* present progress)

Figure 3 shows the remarkable progress in treatment access, 2000–2015, alongside reductions in HIV related deaths and new HIV infections. In several high burden countries, there have been declines in life expectancy due to HIV, and then an increase and reversal of these declines when HIV treatment rolled out [2]. However, the present rates of new infections are not sustainable, declines in new infections among adults have stalled from 2010 to 2015, and require decisive reductions by 75% by 2020. The need to close the gap in treatment coverage and reduce incidence is a major impetus to the review of treatment monitoring for the SDGs, and a focus on gaps in coverage.

**Fig. 4:**
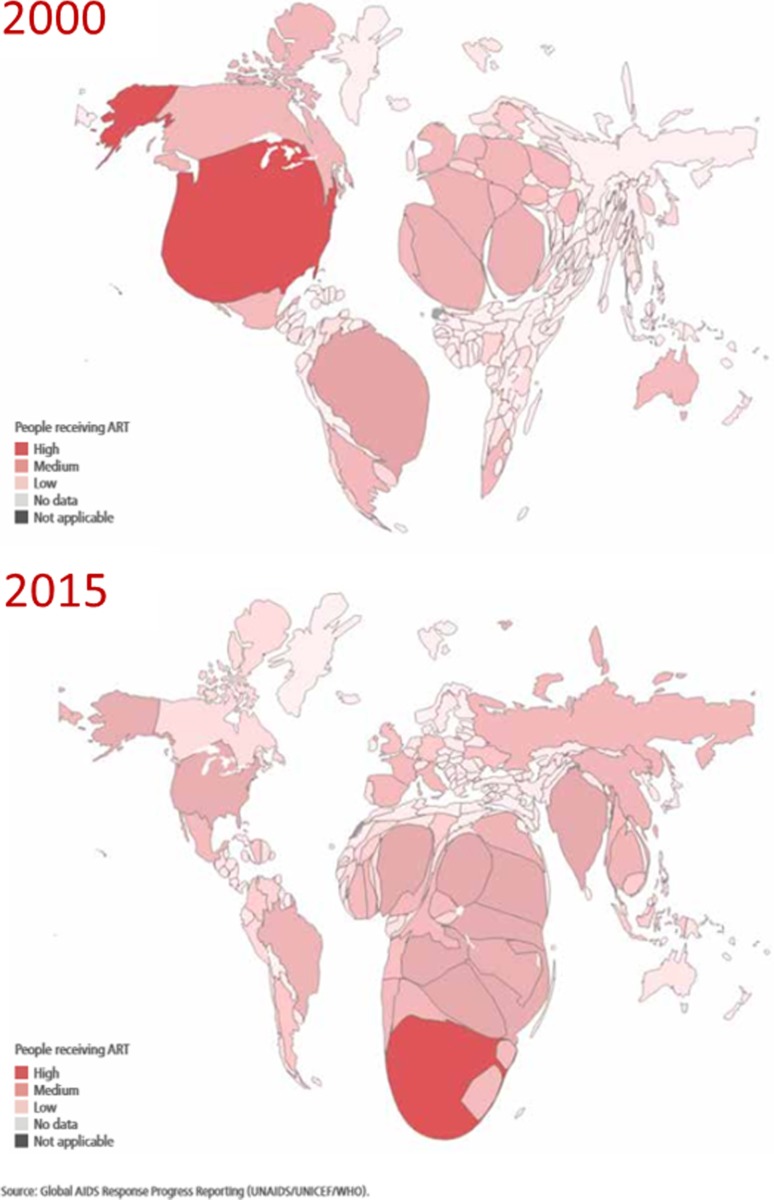
Countries with their size proportional to number of people on treatment in 2000 and 2015

This period showed a transformation in access to treatment globally, and particularly in Africa. Figure 4 shows countries with their size proportional to the number of people on treatment in 2000 and 2015. Africa is now at the centre of the treatment movement, with many of the key innovations, and the largest treatment programs in the world [12]. ART coverage reached 53% among women and 39% among men in Africa, up from less than 1% in 2000, and the number of people dying of HIV-related causes was almost halved in the past decade.

Yet, the limitations of treatment monitoring focused on a single numerical target, are shown by the major challenges that remain. There are gaps along the treatment cascade, in linkage from testing to treatment, retention on treatment, and viral suppression. Therefore the accelerated impact of treatment requires an enhanced approach to treatment monitoring.

## Treatment Monitoring Towards 90, 90, 90 in 2020

The achievement of the treatment goals for 2015 have been followed by the challenge to achieve the 90, 90, 90 goals by 2020, requiring treatment linked to testing, retention and viral suppression, supported by more granular district and patient level reporting systems [11, 13, 14].

The UNAIDS 90, 90, 90 goals and WHO consolidated strategic information guidelines, stress three important developments in treatment monitoring for the period 2015–2020.

First, monitoring treatment numbers is seen as part of the health sector cascade, linking treatment directly to outcomes and impact in terms of incidence and mortality. The consolidated strategic information guidelines provide a consolidated framework for treatment monitoring [10]:

- *Defines ten key global indicators* (and 50 national indicators), agreed and consolidated by partners for HIV and treatment monitoring. These 10 indicators are embedded in the Global Reference list of 100 Core Health Indicators, a standard set of 100 indicators prioritized by the global community to provide concise information on health trends, resulting in a reduction of reporting burden for countries [15].
- *Provides a clear results chain or cascade for treatment*, linking it to prevention, testing and outcomes to promote consolidated analysis of strategic information to better support program decisions and linked services to individuals.
- *Defines data needs* to support disaggregated and granular HIV prevalence data, HIV prevention monitoring for key populations, case and patient monitoring linking individuals to services, and a practical impact evaluation agenda to assess changes in incidence and mortality.
- Supports the *consolidated analysis and use of data*, with cascade analysis to identify gaps in prevention, testing and treatment, and promote the use of data for program improvement.

**Fig. 5:**
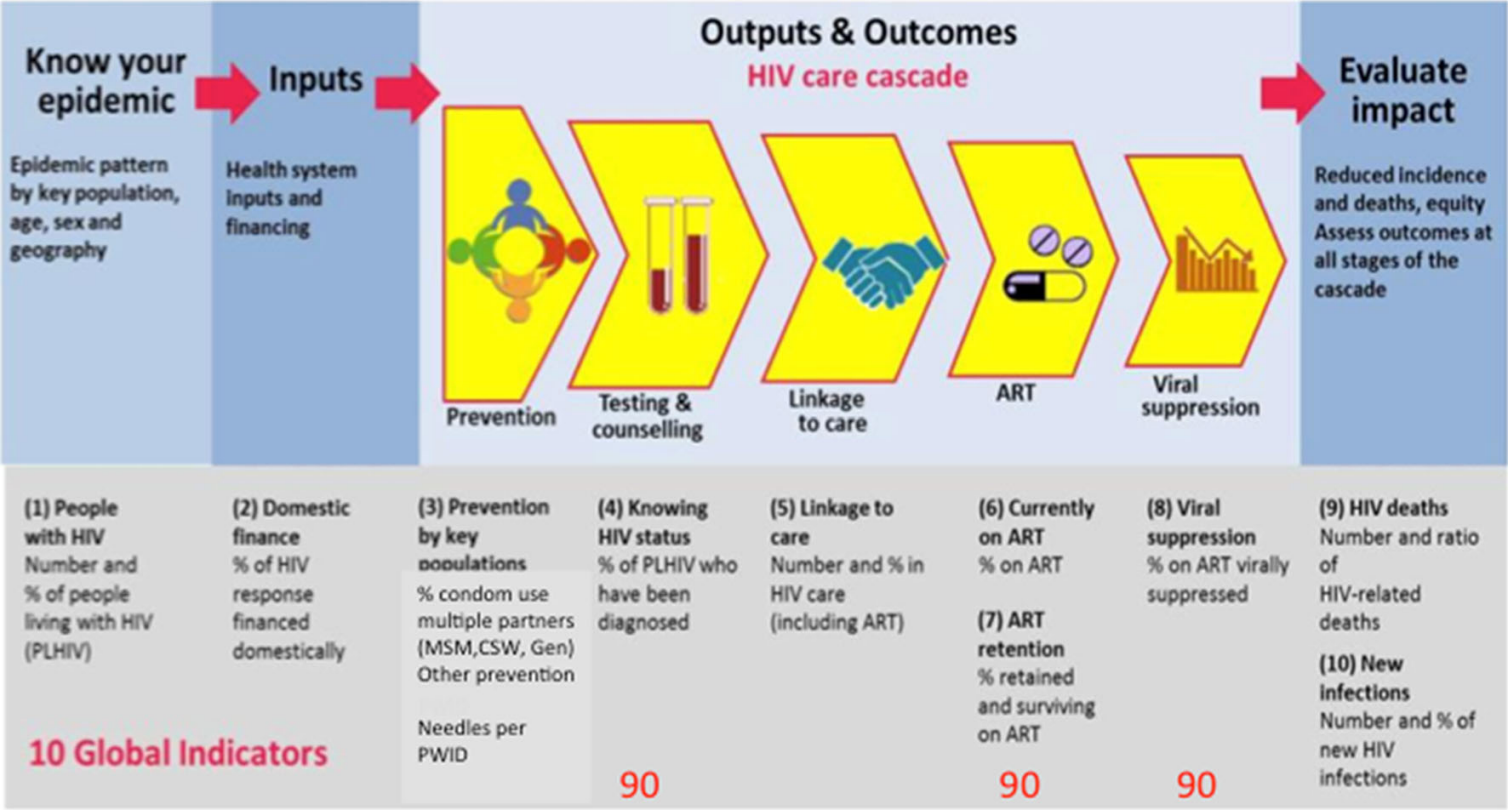
The ten global indicators and 90, 90, 90 targets in the framework of the health sector cascade

The role of treatment monitoring in the health sector cascade, and in relation to the 90, 90, 90 targets is shown in Fig. 5.

Treatment numbers should be assessed in the context of testing, linkage to care, retention and viral suppression [16]. There is a clear coverage denominator of all people living with HIV, which as countries move to Treat All should be used as the basis for target setting.

The health sector cascade starts with prevention and testing, and prevention opportunities should be assessed at each stage of the health sector cascade [17]. They are necessary to achieve decisive declines in HIV incidence.

Secondly, the global monitoring of treatment will require greater investments in sustainable treatment monitoring to support individuals on treatment over the medium term, and ensure longitudinal follow up of retention, viral suppression and analysis of outcomes [10].

There are measurement challenges at each stage of the cascade, in particular for the first and the third 90. Measures of the number of people tested need to be replaced by the percentage of people living with HIV who have been diagnosed and are aware of their status. This highlights the gaps in diagnosis and linkage to treatment, and estimates have been published by UNAIDS/WHO since 2014 in sub Saharan Africa and for the first time across regions in 2015. Measures of viral suppression will require increases in coverage of viral load monitoring, and at present require considerable interpretation. UNAIDS/WHO published the first global and regional estimates of the third 90 in 2016. The second 90, %PLHIV currently on treatment, also requires improved analysis of retention, loss to follow up, and individual level patient monitoring systems to fully understand the gaps identified.

As people move between clinics and are on treatment for 5, 10, 15 years and longer, routinely generated clinical data needs to be transmitted securely and confidentially from points of service delivery to subnational levels for de-duplication, analysis and use.

To achieve this, the new ARV and strategic information guidelines are being translated into individual level patient and case monitoring guidance to:

1. Update and optimise HIV patient monitoring with a minimum standardised dataset, patient card and facility register.
2. Strengthen HIV case based surveillance.
3. Link HIV patient monitoring across ART, TB, MCH and community services.
4. Use data to improve patient care and optimize reporting on national priority indicators along the cascade of prevention, care and treatment.
5. Transition to a unique identifier approach in HIV diagnosis and care and to electronic-based systems at the right level and time, to create an integrated, secure and confidential programme database.

Global treatment monitoring, in line with the SDGs, which highlights data as one of the three cross cutting issues in implementation (alongside institutions and partnerships, in SDG 17), will require greater investments in data systems, disaggregation, and use of data for decisions.

The SDGs will also require that the treatment cascade is evaluated in terms of its contribution to reduced mortality and incidence. A well-defined results chain or cascade framework for treatment monitoring, is also an opportunity for regular, routine impact evaluation of programs. Impact evaluation should aim to make necessary practical adjustments to a program, and identify what is working and what is missing, based on evidence of changes in mortality and incidence.

Despite the major successes of treatment monitoring since 2000, in setting targets, harmonizing reporting, and catalyzing progress, there is no room for complacency. Treatment monitoring systems will need to support large numbers of people on treatment over decades, ensure linkage, retention and viral suppression, and provide aggregated and individual level data for program management in a safe, secure and simplified manner.

The major challenge is well illustrated by the targets for 2020 and 2030 (Fig. 3). The challenge of sustainability relies on decisively reducing new infections. This requires a much more balanced approach to prevention and treatment going forward, measuring and closing the gaps in coverage along the prevention, testing and treatment cascade. There are gaps at each stage of the HIV testing and treatment cascades, and the WHO Global Report in 2016 highlighted the Prevent, Test and Treat approach needed for the decisive reductions in incidence to contribute to sustainable impact, and the spirit and goals of the SDGs [5].

## Conclusion: From Treatment Numbers to Impact

The period 2000–2015 was a remarkable period for treatment monitoring and access. The setting of clear global targets, standardized and harmonized reporting, and accountability mechanisms with regular reporting and analysis at global and country levels, provided a significant focus for global access. The target of 15 million people on treatment was reached before 2015, with Africa closing the relative gap compared to other regions, a distinctive achievement in health and development.

Yet there were also many challenges, changing eligibility and linkage of testing to treatment, retention of people on treatment, and evaluation of outcomes.

In the context of the SDGs, there are three important shifts in treatment monitoring. First, treatment monitoring is shifting from numbers to universal coverage and gaps in a cascade of prevention, testing and treatment services. Second, this shift requires much greater attention to sustainable, individual level, treatment and case monitoring systems for chronic health care. Third, the health sector cascade has a clear results chain, to assess impact in terms of reduced mortality and incidence. This requires a stronger impact evaluation agenda alongside monitoring.

Data now needs to become more granular to link to district, case and patient information systems. The shift from numbers to individual, longitudinal data can contribute further to retention, viral suppression and impact on deaths and new infections, just as the focus on numbers contributed to access. When linked and used at the individual level, data can be an important intervention in its own right to achieve health outcomes.

In line with the SDGs, this will require much greater real time use of treatment data to improve services and programs, disaggregated to the relevant level of accountability and decision, and to consistently close gaps in coverage along a cascade of services. Together with an integrated approach to universal access to prevention, testing and treatment, this will lay the basis for the contribution of treatment to the sustainable impact of the HIV and SDG goals.
